# Characterization of the complete mitochondrial genome of *Balitora ludongensis* (Teleost: Balitoridae) and its phylogenetic analysis

**DOI:** 10.1080/23802359.2020.1773342

**Published:** 2020-06-04

**Authors:** Liyi Shao, Yifei Lin, Tianxu Kuang, Lei Zhou

**Affiliations:** a Joint Laboratory of Guangdong Province and Hong Kong Region on Marine Bioresource Conservation and Exploitation, College of Marine Sciences, South China Agricultural University, Guangzhou, China; b Guangdong Laboratory for Lingnan Modern Agriculture, Guangzhou, China

**Keywords:** *Balitora ludongensis*, mitochondrial genome, phylogenetic analysis

## Abstract

In this study, we assembled the complete mitochondrial genome of *Balitora ludongensis*, based on high-throughput Illumina sequencing. The complete mitochondrial genome of *B. ludongensis* was 16,576 bp in size. It was made up of 13 protein-coding genes, 22 tRNA genes, two rRNA genes, and one control region. Molecular phylogenetic analyses using 13 protein-coding genes revealed the phylogenetic position of *B. ludongensis* in Balitoridae. These data provide basic genetic information for *B. ludongensis*, which is vital for a better understanding of the mitogenomic diversities and evolution of fish species in Balitoridae.

The genus *Balitora* was established by Gray in 1830 (Chen and Tang 2000). There were more than 32 species of *Balitora* reported from southern and southeastern Asia (Fricke et al. 2019). However, to date, no complete mitochondrial genomes have been published in this genus. *Balitora ludongensis* is a hillstream loach living in the headwater of the Pearl River (Liu et al. 2012). In this study, we sequenced, assembled, and annotated the mitochondrial genome of *B. ludongensis* to further explore the phylogenetic evolution of the Balitoridae fishes.

The specimen was collected from Jingxi City, Guangxi Province, China (N106.275, E23.146) in August 2019. It was maintained in South China Agricultural University with the accession no. SCAU-20190805001. The complete mitogenome was sequenced by next-generation sequencing using the Illumina HiSeq2500 instrument (Illumina, Inc., San Diego, CA, USA) with the de novo assembly strategy (Yang et al. 2018).

The complete mitogenome of *B. ludongensis* was 16,576 bp (GenBank Accession number: MT157616) in length, with an A + T content of 54.83% and its base composition: 30.37% A, 24.46% T, 16.4% G, and 28.77% C. Similar to that reported in other vertebrates (Boore 1999; Luo et al. 2017; Wang et al. 2020), the genome has 13 protein-coding genes (PCGs), 22 tRNAs, two rRNAs, and one putative control region (CR). Among all the PCGs, there were 12 genes (nad2, cox1, cox2, atp8, atp6, cox3, nad3, nad4I, nad4, nad5, cob, and nad1) on the heavy strand, while one (nad6) on the light strand. The PCGs used three different start codons, including ATG, GTG, and ATC, and ended with the TAA, TA or T stop codon.

We established a phylogenetic tree based on the mitogenome 13 protein-coding genes from 30 Balitoridae species (Figure 1). Phylogeny showed that the 30 Balitoridae species could be divided into two branches. *Sinogastromyzon*, *Lepturichthys, Jinshaia, Balitora, Pseudohomaloptera, Homaloptera, Homalopteroides, Neohomaloptera and Bhavania* formed branch I, the other genera formed branch II. *Balitora* was the sister group of *(Jinshaia + Lepturichthys + Sinogastromyzon)* with high support value.

**Figure 1.**
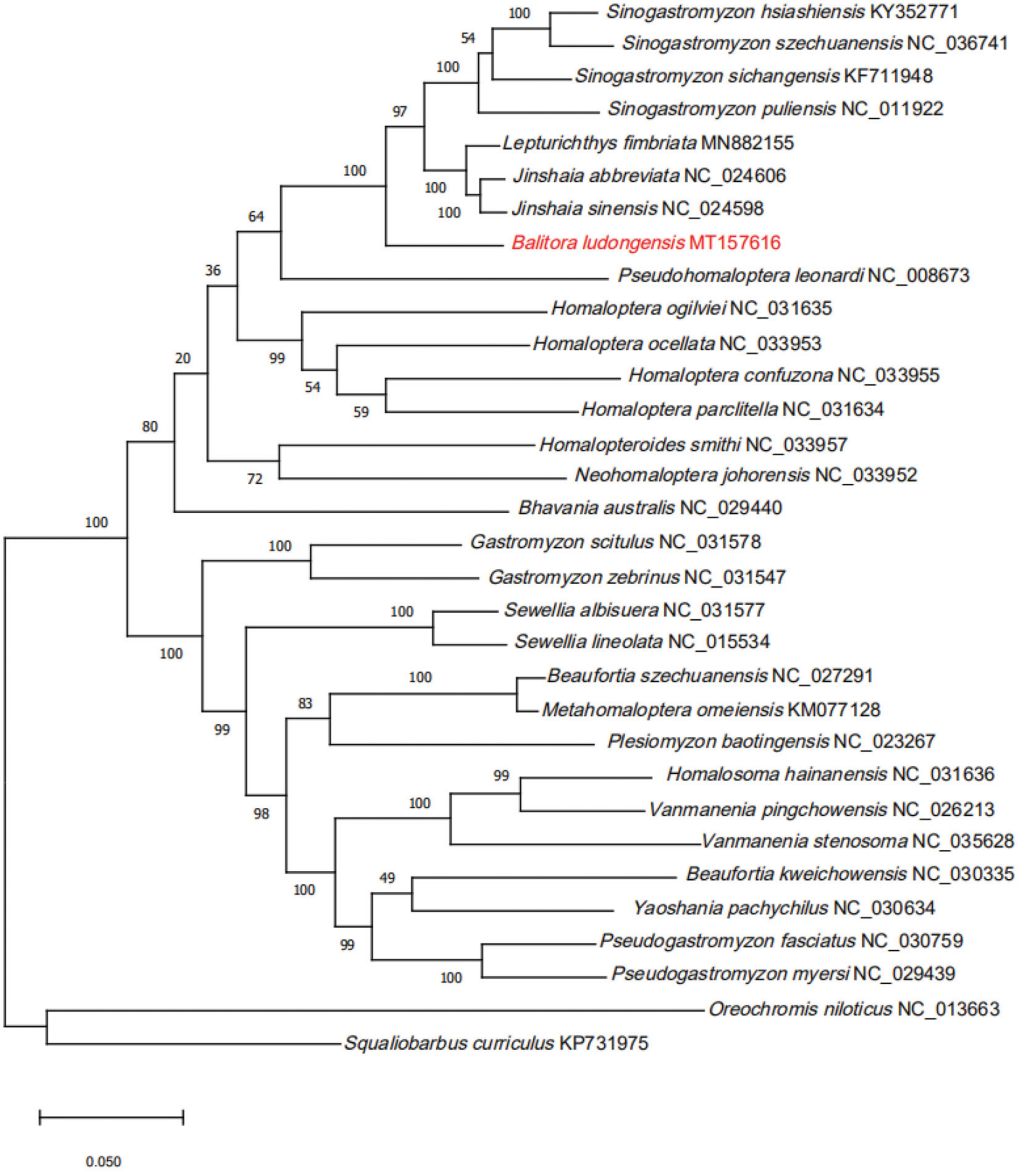
Phylogeny of 30 species within the family Balitoridae based on the Maximum likelihood analysis of 13 mitochondrial protein-coding genes. The support values are shown next to the nodes (1000 replications). *Oreochromis niloticus* (GenBank: NC_013663) and *Squaliobarbus curriculus* (GenBank: KP731975) were included as the outgroup taxon.

In conclusion, we sequenced, annotated, and characterized the complete mitogenome of *B. ludongensis*. Our research results should help elucidate the mitogenomes and phylogeny of the Balitoridae and the species within it, which are essential aspects for future taxonomic, systematic, and genetic studies.

## Data Availability

The data that support the findings of this study are openly available in GenBank of NCBI at https://www.ncbi.nlm.nih.gov, reference number MT157616.
